# Beta cell endoplasmic reticulum stress drives diabetes in the KINGS mouse without causing mass beta cell loss

**DOI:** 10.1111/dme.14962

**Published:** 2022-10-09

**Authors:** Lydia F. Daniels Gatward, Yujin Kim, Aerin Loe, Yiyang Liu, Line Kristensen, Aileen J. F. King

**Affiliations:** ^1^ Department of Diabetes King's College London London UK

**Keywords:** apoptosis, beta cell, beta cell mass, endoplasmic reticulum stress, unfolded protein response

## Abstract

**Aims:**

Beta cell endoplasmic reticulum (ER) stress can cause cellular death and dysfunction and has been implicated in the pathogenesis of diabetes. Animal models of beta cell ER stress are critical in further understanding this and for testing novel diabetes therapeutics. The KINGS mouse is a model of beta cell ER stress driven by a heterozygous mutation in *Ins2*. In this study, we investigated how beta cell ER stress in the KINGS mouse drives diabetes.

**Methods:**

We investigated whether the unfolded protein response (UPR) was activated in islets isolated from male and female KINGS mice and whether this impacted beta cell mass and turnover.

**Results:**

Whilst the UPR was up‐regulated in KINGS islets, with increased protein expression of markers of all three UPR arms, this was not associated with a mass loss of beta cells; beta cell apoptosis rates did not increase until after the development of overt diabetes, and did not lead to substantial changes in beta cell mass.

**Conclusion:**

We propose that the KINGS mouse represents a model where beta cell maladaptive UPR signalling drives diabetes development without causing mass beta cell loss.

**What's new**

- Beta cell endoplasmic reticulum (ER) stress can cause beta cell death and dysfunction and has been implicated in diabetes pathogenesis.
- In this study, we investigated whether beta cell ER stress in a mouse model of diabetes, the KINGS mouse, drives up‐regulation of the unfolded protein response and changes to beta cell turnover and mass.
- We propose that the KINGS mouse represents a model where maladaptive UPR signalling in the beta cells drives diabetes development without causing mass beta cell loss.

## 1 INTRODUCTION

Beta cell endoplasmic reticulum (ER) stress can lead to beta cell dysfunction and death and has been suggested to play an important role in the development of diabetes.^1^, ^2^ Beta cells can respond to ER stress by activating the unfolded protein response (UPR) which encompasses adaptive signalling pathways to resolve ER stress and restore homeostasis.^3^ Protein kinase R‐like endoplasmic reticulum kinase (PERK), inositol requiring enzyme 1 (IRE1) and activating transcription factor 6 (ATF6) act as ER stress sensors residing within the ER membrane. In an unstressed state, these sensors are kept inactive through engagement with the protein chaperone, binding immunoglobulin protein (BiP). In an ER‐stressed state, BiP is recruited to facilitate protein folding in the ER lumen, allowing the ER stress sensors to become activated and initiate UPR adaptive signalling. This signalling restores ER homeostasis in several ways including through attenuating global mRNA translation, increasing the gene expression of protein chaperones as well as through increasing gene expression of ER‐associated protein degradation components.

Adaptive UPR signalling is critical in maintaining a functional beta cell population.^4^, ^5^ It maintains protein homeostasis in the face of mild ER stress resulting from transient and physiologically normal increases in insulin processing. It has also been implicated in beta cell proliferation,^6^ insulin secretory function,^7^ protection from apoptosis and maintenance of beta cell identity in response to stressful stimuli.^8^, ^9^ Deletion of many components of the UPR has been associated with beta cell demise and the consequential development of diabetes.^10^, ^11^, ^12^ In line with this, a failure of the adaptive UPR to be mounted has also been proposed to play a role in diabetes pathogenesis.

On the other hand, activation of maladaptive UPR signalling which occurs when ER stress is too robust to be resolved through adaptive signalling can lead to beta cell loss.^1^, ^13^ Chronic signalling through PERK leads to the activation of proapoptotic C/EBP homologous protein (CHOP). Likewise, chronic IRE1 signalling drives the phosphorylation and activation of the pro‐apoptotic protein c‐Jun N‐terminal kinase (JNK). Chronic IRE1 signalling also drives global mRNA degradation through regulated IRE1 dependant decay which when sustained can lead to a loss of cellular identity and apoptosis. Maladaptive UPR signalling resulting from irremediable ER stress has been associated with beta cell loss and the development of diabetes in the Akita mouse^14^ and the Munich mouse models.^15^, ^16^

The KINGS mouse is a newly characterised mouse model of diabetes caused by beta cell ER stress which harbours a single nucleotide polymorphism in *Ins2*.^17^ In this study, we investigated whether the KINGS mutation drives activation of the UPR in beta cells and the impact of this on beta cell mass and turnover. We propose that the KINGS mouse represents a model where maladaptive UPR signalling in the beta cells drives diabetes development without causing mass beta cell loss.

## 2 RESEARCH DESIGN AND METHODS

### 2.1 Animals

Mice were housed in individually ventilated cages in groups of up to five with ad libitum access to water and standard rodent diet 20 chow (Picolab). Animals were kept on a 7 AM–7 PM light–dark cycle. Heterozygous KINGS and wildtype mice were maintained through in‐house conventional breeding. All in vivo procedures were approved by our institution's ethics committee and performed under license in accordance with the U.K. Home Office Animals (Scientific Procedures) Act 1986 with 2012 amendments.

### 2.2 Genotyping

Ear clips were digested for DNA extraction using DNA lysis buffer (10% 10× Gitschier buffer, 0.5% Triton X‐100, 1% β‐mercaptoethanol, 2% 50 μg/ml proteinase K). Kompetitive allele‐specific PCR (KASP; LGC) was used to determine the presence of the KINGS mutation as previously described.^17^

### 2.3 Blood glucose measurements and glucose tolerance test

Non‐fasted blood glucose concentrations in mice were measured using an Accu‐Chek glucose meter after a blood droplet was generated via a needle‐prick to the end of the tail (6–15 mice per group). For the glucose tolerance test, mice were fasted for 6 h after which basal blood glucose concentrations were measured. Two grams per kilogram glucose was subsequently injected intraperitoneally, and blood glucose concentrations were measured at 15, 30, 60, 90 and 120 min post‐injection (7–11 mice per group).

### 2.4 Islet isolation

Islets were isolated from mice culled by cervical dislocation through collagenase digestion as previously described.^17^ Briefly, pancreases were perfused with 1 mg/ml collagenase solution via injection through the bile duct, digested at 37°C for 10‐min and islets separated using a Histopaque‐1077 density gradient. Islets were washed before culture overnight at 37°C in RPMI medium (11.1 mM glucose concentration) supplemented with 10% fetal bovine serum and 1% penicillin/streptomycin.

### 2.5 Western blotting

Islets were lysed with radioimmunoprecipitation assay buffer supplemented with a protease and phosphatase inhibitor cocktail. Ten micrograms of protein per sample was subjected to sodium dodecyl sulphate‐polyacrylamide gel electrophoresis and transferred to a polyvinylidene fluoride membrane (PVDF). The PVDF membrane was sequentially incubated with antibodies toward ER stress markers; rabbit anti‐BiP (1:1000; Cell Signalling Technology), mouse anti‐beta‐actin (1:5000; Santa Cruz Biotechnology), rabbit anti‐XBP1s (1:1000; Cell Signalling Technology), rabbit anti‐p‐eIF‐2‐alpha (1:1000; Cell Signalling Technology), mouse anti‐CHOP (1:100; Santa Cruz Biotechnology) and mouse anti‐ATF6 (1:500; Bio‐Techne), and subsequently HRP‐conjugated anti‐mouse and anti‐rabbit antibodies (1:2500; Santa Cruz Biotechnology) with washing between incubations but without stripping. Densitometry analysis was performed in ImageJ, and peak percentages were normalised to the beta actin loading control. The experimental unit was pooled islets from 2 to 3 mice. Each experiment was carried out using 4–6 independent experimental units.

### 2.6 Sample processing for immunofluorescent microscopy

Mice were killed by cervical dislocation and pancreases excised and fixed for 48 h in 4% phosphate‐buffered formalin. Tissues were wax embedded using an automated tissue processer (Leica), cut into 5 μM sections and mounted onto microscope slides.

### 2.7 Beta cell mass

Three to four sections spanning the entire pancreas and ~580 μM apart were dewaxed, blocked in blocking buffer (5% milk, 0.1% Triton‐X, 0.5% goat serum) and incubated overnight at 4°C with a guinea‐pig anti‐insulin antibody at 1:200 (Abcam). After washing, sections were subsequently incubated with a secondary anti‐guinea pig antibody (Alexa Fluor 488, 1:100; Jackson ImmunoResearch) and 4′,6‐diamidino‐2‐phenylindole (DAPI, 1:500) for 1‐h at room temperature. Whole pancreatic sections were imaged using a NanoZoomer S60 fluorescence and brightfield slide scanner (Hamamatsu) with a 20× objective. Forty‐two mice were used to study beta cell mass (3–5 animals per group; three ages studied in both male and female wildtype and KINGS mice). Beta cell area and whole pancreatic area were analysed semi‐automatically in FijiImageJ.

### 2.8 Beta cell apoptosis

Beta cell apoptosis was investigated through immunofluorescent TUNEL staining using the Apoptag Fluorescein In situ Apoptosis Detection Kit (Merck) per the manufacturer's guidelines. For identification of beta cells, slides were subsequently incubated with a primary guinea pig antibody toward insulin (Dako) overnight at 4°C and then with a secondary anti‐guinea pig antibody (Alexa Fluor 594, 1:100; Jackson ImmunoResearch) and DAPI (1:500). Thirty‐six animals were used to study apoptosis (three animals per group; three ages studied in both male and female wildtype and KINGS mice). Islets from one pancreatic section (441–3618 beta cells) per animal were imaged at 10 or 20× magnification on a Nikon Eclipse TE 2000‐U microscope. All image analysis was carried out manually and in a blinded fashion in ImageJ.

### 2.9 Beta cell proliferation and islet area

Pancreatic sections were de‐waxed and underwent citric acid heat‐mediated antigen retrieval. Sections were then blocked in blocking buffer (5% milk, 0.1% Triton‐X, 0.5% goat serum) and incubated overnight at 4°C with a guinea‐pig anti‐insulin antibody (Dako and Abcam; 1:200) and rabbit anti‐Ki‐67 antibody at 1:100 (Abcam). After washing, sections were subsequently incubated with a secondary anti‐guinea pig antibody (Alexa Fluor 488, 1:100; Jackson ImmunoResearch), anti‐rabbit antibody (Alexa Fluor 594, 1:100; Jackson ImmunoResearch) and DAPI (1:500) for 1 h at room temperature. Islets were imaged at 10–30× magnification on a Nikon Eclipse TE 2000‐U microscope. Forty‐four animals were used to study beta cell proliferation (3–5 animals per group; three ages studied in both male and female wildtype and KINGS mice). All islets from 1 to 2 pancreatic sections (665–3199 beta cells per mouse) were imaged in total. For islet area, 44 animals were used (3–5 animals per group), and 65–161 islets were analysed per group. In a separate experiment, haematoxylin and eosin staining was used to stain pancreatic sections, and islet area was analysed; 36 animals in total were used (three animals per group), and 63–137 islets were analysed per group. All image analysis was carried out manually and in a blinded fashion in ImageJ.

### 2.10 Statistical analysis

Two‐way analysis of variance (ANOVA) with Holm‐Sidak post hoc tests were used to compare multiple groups using SigmaPlot version 14.5 software. *p* < 0.05 was considered statistically significant, and all data are represented as mean ± standard error of the mean. Graphs were created using GraphPad Prism version 9.

## 3 RESULTS

### 3.1 KINGS male mice develop diabetes from 5 weeks

**FIGURE 1:**
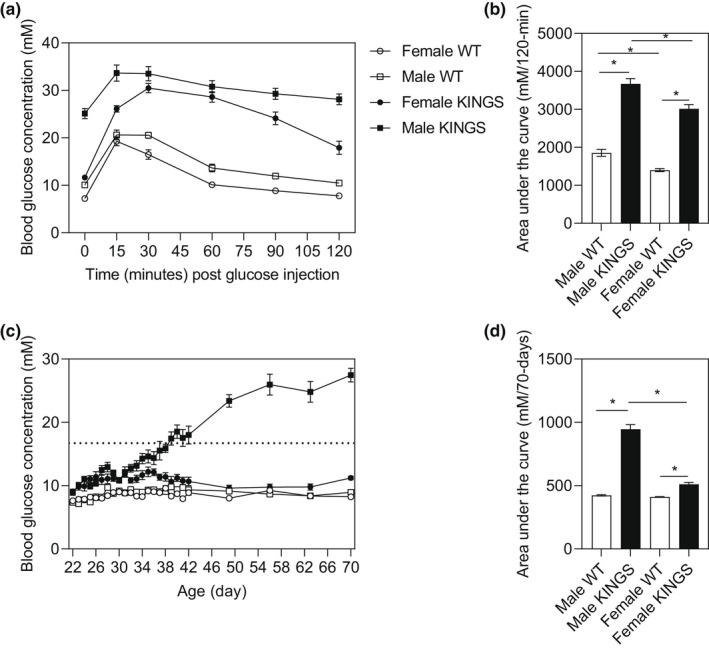
Glucose intolerance and sexual dimorphic diabetes phenotype were confirmed in the KINGS mouse. (a) Blood glucose concentrations of 10‐week‐old male and female KINGS mice and their wildtype littermates after intraperitoneal glucose administration. (b) Area under the curve for glucose tolerance tests, *n* = 6–15. (c) Daily non‐fasted blood glucose concentrations from 22 to 42 days and weekly blood glucose concentrations from 42 to 70 days, in KINGS males and females and their wildtype littermates. The horizontal dotted line represents the threshold for over diabetes (16.7 mM). (d) Area under the curve for non‐fasted blood glucose concentrations measured over 70 days in male and female KINGS mice and wildtype littermates, *n* = 7–11. Two‐way ANOVA with Holm‐Sidak post hoc, *p < 0.05. ANOVA, analysis of variance.

Glucose intolerance was confirmed in 10‐week male and female KINGS mice (Figure 1). Additionally, male KINGS blood glucose concentrations were increased by 27 days of age compared to weaning at 22 days. This progressively worsened and overt diabetes (>16.7 mM) occurred between 5 and 6 weeks. In contrast, female KINGS mice remained normoglycemic (<11.1 mM) and blood glucose concentrations were significantly different between the sexes by 34 days (p < 0.05, two‐way repeated measures ANOVA). Subsequent experiments were performed in mice at 4, 10 and 20 weeks of age as these timepoints encompass disease progression in the KINGS males (4 weeks: prior to overt diabetes, 10 weeks: overt diabetes, 20 weeks: chronic diabetes).

### 3.2 The UPR is activated in KINGS islets

**FIGURE 2:**
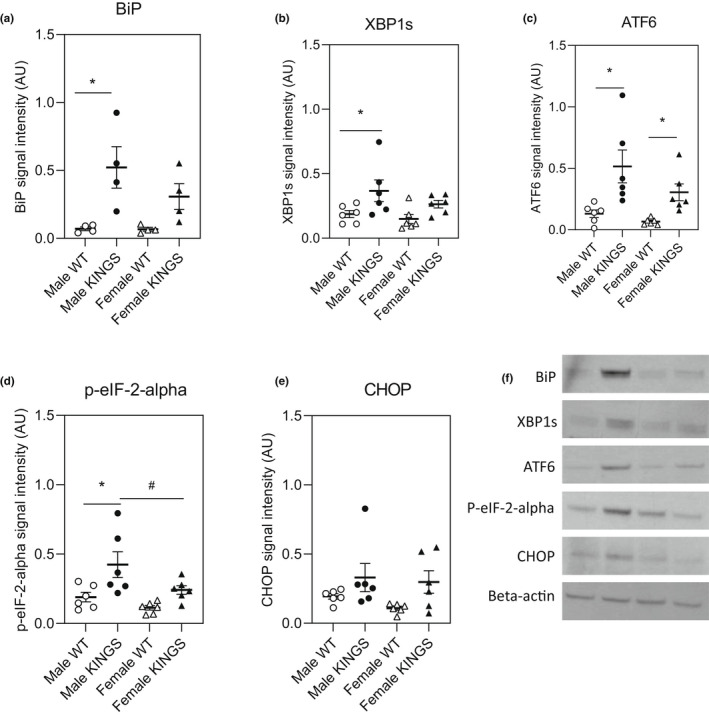
Up‐regulation of UPR pathways in the KINGS mouse islets. (a–e) Levels of ER stress markers were investigated in islets isolated from 10‐week‐old mice. (f) Representative Western blot images. Wildtype (WT) male islets (Lane 1), KINGS male islets (Lane 2), WT female islets (Lane 3), KINGS female islets (Lane 4). *n* = 4–6, two‐way ANOVA with Holm‐Sidak's post hoc, *p < 0.05 KINGS versus wildtype, ^#^ *p* < 0.05 KINGS males versus KINGS female. ANOVA, analysis of variance; ER, endoplasmic reticulum; UPR, unfolded protein response.

Ten‐week male KINGS islets showed increased expression of BiP, XBP1s, ATF6 and phosphorylated eIF‐2‐alpha compared to wildtype islets (Figure 2). Similarly, expression of these UPR markers tended to be higher in 10‐week female KINGS islets compared to female wildtype islets, but this only reached significance for ATF6. A sex difference was seen in phosphorylated‐eIF‐2‐alpha with greater expression seen in male KINGS islets compared to female KINGS islets. This pattern was also noted for BiP, XBP1s and ATF6 although not at a statistically significant level. CHOP expression in islets from both males and females KINGS mice was varied, with a few animals in each group showing considerably more expression than wildtype but the mean signal was comparable in male KINGS versus female KINGS mice. Islets from 4‐week‐old mice showed a similar pattern of expression of ER stress and UPR markers to 10‐week‐old mice, with the exception of XBP1s. ATF6 was significantly increased in both male and female 4‐week KINGS islets compared to wildtype and phosphorylated‐eIF2‐alpha was also increased but this only reached significance in the males (Figure S2).

### 3.3 Beta cell apoptosis is not associated with diabetes development but does occur after chronic diabetes

**FIGURE 3:**
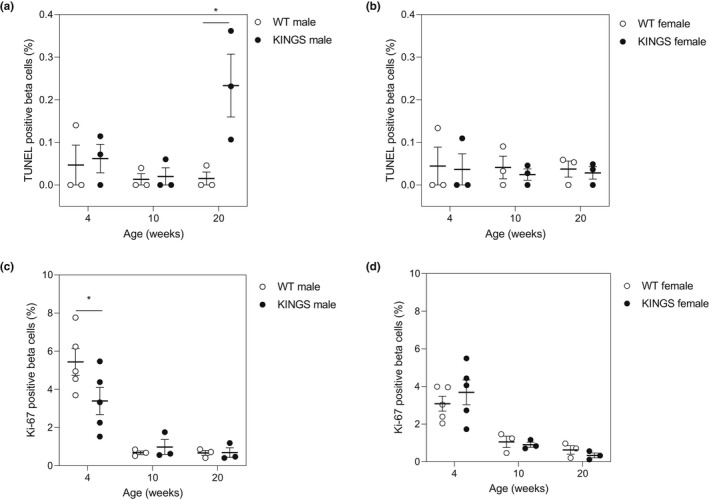
Beta cell apoptosis and proliferation in the male and female KINGS mice. (a, b) Percent total analysed beta cells that were TUNEL positive in (a) WT and KINGS male mice and (b) WT and KINGS female mice. (c, d) Percent total analysed beta cells that were Ki‐67 positive in (c) WT and KINGS male mice and (d) WT and KINGS female mice. *n* = 3–5, two‐way ANOVA with Holm‐Sidak post hoc test, *p < 0.05. ANOVA, analysis of variance; WT, wildtype.

To investigate whether beta cell apoptosis was driving diabetes development in the KINGS mice, beta cell apoptosis was determined at 4, 10 and 20 weeks (Figure 3). Beta cell apoptosis was unchanged in KINGS males and KINGS females compared to wildtype mice at 4 and 10 weeks. However, whilst average beta cell apoptosis also remained unchanged in female KINGS mice at 20 weeks, it increased in male KINGS mice to 15‐fold that of wildtype males.

### 3.4 Beta cell proliferation

Activation of the ATF6 arm of the UPR has previously been associated with increased beta cell proliferation; however, maladaptive UPR signalling supresses proliferation. Since we found ATF6 pathway activation in KINGS islets, we investigated beta cell proliferation in these mice (Figure 3). Beta cell proliferation in all groups was highest at 4 weeks of age and dropped significantly from 10 weeks onwards. In female KINGS mice, the rate of beta cell proliferation was similar to wildtype mice at all ages. However, a reduction in beta cell proliferation was observed in male KINGS mice compared to wildtype mice at 4 weeks, an effect which was lost by 10 and 20 weeks.

### 3.5 Islet size and beta cell mass is unchanged in the KINGS mouse

**FIGURE 4:**
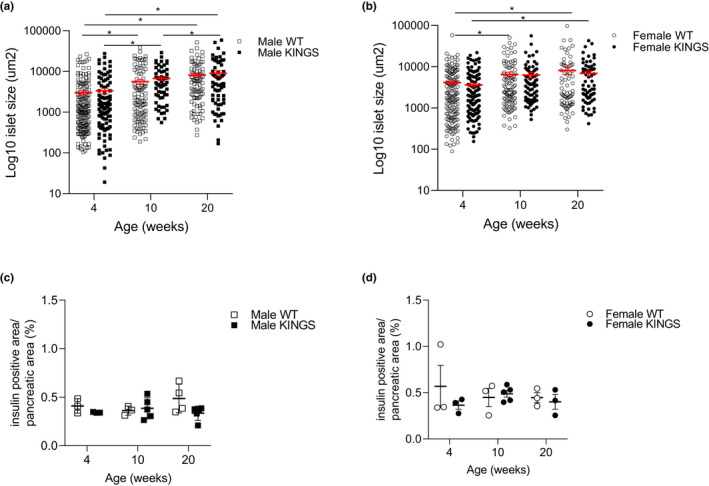
Islet size and beta cell mass is unchanged in the KINGS mouse. Islet size was measured after immunofluorescent staining of pancreatic sections for insulin in (a) WT and KINGS male mice and (b) WT and KINGS female mice at 4, 10, 20 weeks of age. Islets pooled from *n* = 3–5 mice, two‐way ANOVA with Holm‐Sidak post hoc, *p < 0.05. Percentage insulin‐positive area/pancreatic area was measured in male (c) and female (d) KINGS and WT mice at 4, 10 and 20 weeks of age. No significant differences were found between groups, *n* = 3–5. ANOVA, analysis of variance; WT, wildtype.

Since beta cell apoptosis and proliferation rates are low, it cannot be ruled out that statistically non‐significant but biologically relevant changes could impact on beta cell mass. We therefore investigated islet area and beta cell mass in the KINGS mice (Figure 4). A significant increase in islet area with age was observed in all groups. However, no differences in islet area were found between KINGS and wildtype mice in male or female mice at any age. Insulin content has previously been reported to be decreased in the KINGS male and female mice which may impact insulin staining. Therefore, haematoxylin and eosin staining was also used to stain islets and islet area was measured in this way (Figure S1). Similar findings were observed; however, a mild reduction in islet size was found at 10 weeks only in KINGS males.

As beta cell mass could be affected by islet number as well as islet size, we investigated insulin area/total pancreatic area at 4, 10 and 20 weeks of age (Figure 4). In line with no change in islet area in the KINGS mice, beta cell percentage of total pancreatic area was unchanged in male and females between KINGS and wildtype groups at 4, 10 and 20 weeks.

## 4 DISCUSSION

In this study, we investigated whether the UPR was up‐regulated in a novel model of beta cell ER stress, the KINGS mouse, and whether this drives changes in beta cell turnover and beta cell mass. We confirmed that both male and female KINGS mice exhibit marked glucose intolerance compared to wildtype littermates. Moreover, in line with previous studies, male KINGS mice reliably develop overt diabetes by 5–6 weeks of age whilst female KINGS mice remain normoglycemic.^17^

Expression of the general ER stress marker, BiP, was increased in 4‐week male and female KINGS islets suggesting that beta cell ER stress precedes the development of diabetes. In addition, with the exception of XBP1s, there were signs of UPR activation in both sexes at 4 weeks with significant increases in ATF6 observed in males and female KINGS islets, increased phosphorylated eIF‐2‐alpha in male islets and a trend for increased phosphorylated eIF‐2‐alpha in female islets (*p* = 0.066). Overall, these results indicate that KINGS beta cells show clear signs of ER stress at 4 weeks and the UPR has been fully or partially activated at this age. In 10‐week‐old male KINGS islets, expression of XBP1s (which is indicative of IRE1 pathway activation), full‐length ATF6 (indicative of ATF6 pathway activation) and phosphorylated eIF‐2‐alpha (indicative of PERK pathway activation) were all up‐regulated. Male KINGS mice are overtly diabetic at this age, indicating a failure of the UPR to adequately resolve beta cell ER stress to avoid the onset of hyperglycaemia. Female KINGS islets showed a similar pattern of UPR expression, but only ATF6 expression was significantly increased compared to wildtype. Female KINGS mice are glucose intolerant but do not develop overt diabetes indicating that there is either a partial ability of female islets to adapt to ER stress or the ER stress is less than in males. Indeed, female KINGS islets tended to have lower levels UPR markers at 4 and 10 weeks of age, as well as lower and more variable levels of BiP.

The highest and most consistent increases in ER stress and UPR pathways were seen in male KINGS islets at 10 weeks of age. This could be in‐part explained by the effect of hyperglycaemia. Indeed, high glucose concentrations have been shown to induce beta cell ER stress both in vivo and in vitro.^18^, ^19^ However, we have previously rescued male KINGS mice from hyperglycaemia through islet transplantation and found that despite a small improvement in glucose‐stimulated insulin secretion and insulin content, insulin content was still ~80% reduced compared to wildtype islets suggesting that beta cell dysfunction is not solely driven by the effects of high glucose concentrations.^17^ In addition, the islets in the current study were cultured overnight before use, which may have allowed male islets to recover somewhat from the hyperglycaemic environment. Therefore, although the hyperglycaemic environment may contribute to the ER stress seen in male KINGS islets at 10 weeks, it is not the sole cause.

To investigate how female islets may be adapting to the ER stress caused by the *Ins2* ^G32S^ mutation, markers of beta cell turnover were studied. It has been previously suggested that mild ER stress can promote beta cell proliferation via activation of the ATF6 pathway, whereas as chronic ER stress can lead to beta cell apoptosis.^6^, ^9^ Other animal models of beta cell ER stress such as the Akita mouse have shown substantially reduced beta cell mass, and it has been suggested this is a main contributory factor to hyperglycaemia.^20^ Despite activation of the ATF6 pathway, male KINGS mice had a reduction in beta cell proliferation at 4 weeks. Interestingly, the Akita mutation has also been associated with a reduction in beta cell proliferation in neonatal mice, and whilst this effect is lost by 2–3 months when proliferation rates are comparable to wildtype mice, this was proposed to drive the substantial loss in beta cell mass seen in later life.^20^

Up‐regulation of UPR components has also been associated with beta cell apoptosis, and this can be mediated by CHOP downstream of the ATF6 and PERK pathways and JNK downstream of the IRE1 pathway. We only detected increased apoptosis levels in 20‐week‐old male KINGS mice, suggesting that diabetes development is not mediated by beta cell apoptosis. The apoptosis seen in these older male mice may be a result of chronic hyperglycaemia. Indeed, hyperglycaemia exacerbates ER‐stress and beta cell apoptosis.^18^, ^19^, ^21^, ^22^, ^23^ In the Akita mouse, CHOP‐mediated beta cell apoptosis has been proposed to cause diabetes development, and indeed, deletion of CHOP in this model delays diabetes onset.^16^ CHOP expression was variable in both male and female KINGS islets, and we failed to detect any differences compared to wildtype islets or any sex differences at 10 weeks. Our results indicate beta cell apoptosis in the KINGS male mice is a consequence of hyperglycaemia rather than a cause. However, it is important to note that not all studies have reported an increase in beta cell apoptosis in the Akita mouse highlighting that low‐level beta cell apoptosis, which may be biologically relevant, can be difficult to detect.^20^ Beta cell proliferation rates were similarly low, and thus, we investigated islet size and insulin area as a proportion of total pancreatic area.

No significant differences between KINGS and wildtype mice for either islet size or insulin area/pancreatic area were detected when pancreas sections were stained for insulin, and only a mild reduction in islet size in 10‐week‐old male KINGS mice was detected when sections were stained with haematoxylin and eosin. Interestingly, we saw a trend for reduced beta cell mass at 4 and at 20 weeks in KINGS male mice, although this did not reach significance, these results are in line with the findings of reduced proliferation at 4 weeks and enhanced beta cell apoptosis at 20 weeks, and so it is possible that there is a subtle reduction in beta cell mass in this model. This contrasts starkly to findings in the Akita mouse where at 2–3 months a ≥60% reduction in beta cell mass is observed.^20^, ^24^

Diabetes in the KINGS model is likely predominantly driven by ER stress‐mediated beta cell dysfunction rather than beta cell death. In line with this, we have previously shown impaired glucose‐stimulated insulin secretion and insulin content in male and female islets isolated from 10‐week KINGS mice, and this impairment is considerably more severe in male KINGS islets which may explain sex differences in phenotype. We propose that the KINGS mouse represents a model where beta cell ER stress drives maladaptive UPR signalling and the development of diabetes in the absence of mass beta cell loss.

## FUNDING INFORMATION

This work was funded by the Medical Research Council Doctoral Training Programme. For the purpose of open access, the author has applied a Creative Commons Attribution (CC BY) licence to any Author Accepted Manuscript version arising.

## CONFLICT OF INTEREST

No potential conflicts of interest relevant to this article were reported.

## Supporting information


Figure S1
Figure S2

## Data Availability

The data that support the findings of this study are available from the corresponding author upon reasonable request

## References

[1] Shrestha N , de Franco E , Arvan P , Cnop M . Pathological β‐cell endoplasmic reticulum stress in type 2 diabetes: current evidence. Front Endocrinol. 2021;12:650158. doi:10.3389/fendo.2021.650158 PMC8101261 33967960

[2] Eizirik DL , Cardozo AK , Cnop M . The role for endoplasmic reticulum stress in diabetes mellitus. Endocr Rev. 2008;29(1):42‐61. doi:10.1210/er.2007-0015 18048764

[3] Ron D , Walter P . Signal integration in the endoplasmic reticulum unfolded protein response. Nat Rev Mol Cell Biol. 2007;8(7):519‐529. doi:10.1038/nrm2199 17565364

[4] Lenghel A , Gheorghita AM , Vacaru AM , Vacaru AM . What is the sweetest UPR flavor for the β‐cell? That is the question. Front Endocrinol. 2021;11:614123. doi:10.3389/fendo.2020.614123 PMC7891099 33613449

[5] Rabhi N , Salas E , Froguel P , Annicotte JS . Role of the unfolded protein response in β cell compensation and failure during diabetes. J Diabetes Res. 2014;2014:1‐11. doi:10.1155/2014/795171 PMC4000654 24812634

[6] Sharma RB , Arvan P , Alonso LC , et al. Insulin demand regulates b cell number via the unfolded protein response find the latest version: insulin demand regulates β cell number via the unfolded protein response. J Clin Invest. 2015;125(10):3831‐3846. doi:10.1172/JCI79264.15 26389675 PMC4607122

[7] Lee AH , Heidtman K , Hotamisligil GS , Glimcher LH . Dual and opposing roles of the unfolded protein response regulated by IRE1α and XBP1 in proinsulin processing and insulin secretion. Proc Natl Acad Sci U S A. 2011;108(21):8885‐8890. doi:10.1073/pnas.1105564108 21555585 PMC3102350

[8] Lee K , Chan JY , Liang C , et al. XBP1 maintains beta cell identity, represses beta‐to‐alpha cell transdifferentiation and protects against diabetic beta cell failure during metabolic stress in mice. Diabetologia. 2022;65:984‐996. doi:10.1007/s00125-022-05669-7 35316840 PMC9076738

[9] Sharma RB , Landa‐Galván HV , Alonso LC . Living dangerously: protective and harmful ER stress responses in pancreatic β‐cells. Diabetes. 2021;70(11):2431‐2443. doi:10.2337/dbi20-0033 34711668 PMC8564401

[10] Zhang P , McGrath B , Li S , et al. The PERK eukaryotic initiation factor 2α kinase is required for the development of the skeletal system, postnatal growth, and the function and viability of the pancreas. Mol Cell Biol. 2002;22(11):3864‐3874. doi:10.1128/mcb.22.11.3864-3874.2002 11997520 PMC133833

[11] Delepine M , Nicolino M , Barrett T , Golamaully M , Lathrop MG , Julier C . EIF2AK3, encoding translation initiation factor 2‐α kinase 3, is mutated in patients with Wolcott‐Rallison syndrome. Nat Genet. 2000;25:406‐409. 10932183 10.1038/78085

[12] Hassler JR , Scheuner DL , Wang S , et al. The IRE1α/XBP1s pathway is essential for the glucose response and protection of β cells. PLoS Biol. 2015;13(10):1‐22. doi:10.1371/journal.pbio.1002277 PMC4607427 26469762

[13] Ghosh R , Colon‐Negron K , Papa FR . Endoplasmic reticulum stress, degeneration of pancreatic islet β‐cells, and therapeutic modulation of the unfolded protein response in diabetes. Mol Metab. 2019;27:S60‐S68. doi:10.1016/j.molmet.2019.06.012 PMC6768499 31500832

[14] Yoshioka M , Kayo T , Ikeda T , Koizumi A . A novel locus, Mody4, distal to D7Mit189 on chromosome 7 determines early‐onset NIDDM in nonobese C57BL/6 (Akita) mutant mice. Diabetes. 1997;46(5):887‐894. doi:10.2337/diabetes.46.5.887 9133560

[15] Herbach N , Rathkolb B , Kemter E , et al. Dominant‐negative effects of a novel mutated ins2 allele causes early‐onset diabetes and severe beta‐cell loss in Munich mutant mice. Diabetes. 2007;56(5):1268‐1276. doi:10.2337/db06-0658.ENU 17303807

[16] Oyadomari S , Koizumi A , Takeda K , et al. Targeted disruption of the Chop gene delays endoplasmic reticulum stress‐mediated diabetes. J Clin Investig. 2002;109(4):525‐532. doi:10.1172/JCI0214550 11854325 PMC150879

[17] Austin ALF , Daniels Gatward LF , Cnop M , et al. The KINGS Ins2+/G32S mouse: a novel model of β‐cell endoplasmic reticulum stress and human diabetes. Diabetes. 2020;69(12):2667‐2677. doi:10.2337/db20-0570 32994272 PMC7679781

[18] Tang C , Koulajian K , Schuiki I , et al. Glucose‐induced beta cell dysfunction in vivo in rats: link between oxidative stress and endoplasmic reticulum stress. Diabetologia. 2012;55(5):1366‐1379. doi:10.1007/s00125-012-2474-8 22396011

[19] Elouil H , Bensellam M , Guiot Y , et al. Acute nutrient regulation of the unfolded protein response and integrated stress response in cultured rat pancreatic islets. Diabetologia. 2007;50(7):1442‐1452. doi:10.1007/s00125-007-0674-4 17497122

[20] Riahi Y , Israeli T , Yeroslaviz R , et al. Inhibition of mTORC1 by ER stress impairs neonatal β‐cell expansion and predisposes to diabetes in the Akita mouse. Elife. 2018;7:1‐25. doi:10.7554/eLife.38472 PMC6294551 30412050

[21] Qian B , Wang H , Men X , et al. TRIB3 is implicated in glucotoxicity‐ and oestrogen receptor‐stress‐induced β‐cell apoptosis. J Endocrinol. 2008;199(3):407‐416. doi:10.1677/JOE-08-0331 18818302

[22] Maedler K , Schulthess FT , Bielman C , et al. Glucose and leptin induce apoptosis in human β‐ cells and impair glucose‐stimulated insulin secretion through activation of c‐Jun N‐terminal kinases. FASEB J. 2008;22(6):1905‐1913. doi:10.1096/fj.07-101824 18263705

[23] Laybutt DR , Preston AM , Åkerfeldt MC , et al. Endoplasmic reticulum stress contributes to beta cell apoptosis in type 2 diabetes. Diabetologia. 2007;50(4):752‐763. doi:10.1007/s00125-006-0590-z 17268797

[24] Winnay JN , Dirice E , Liew CW , Kulkarni RN , Kahn CR . P85α deficiency protects β‐cells from endoplasmic reticulum stress‐induced apoptosis. Proc Natl Acad Sci U S A. 2014;111(3):1192‐1197. doi:10.1073/pnas.1322564111 24395790 PMC3903202

