# Supplementary material for: Beta cell endoplasmic reticulum stress drives diabetes in the KINGS mouse without causing mass beta cell loss
Source: Diabet Med. 2022 Oct 9;39(12):e14962. doi: 10.1111/dme.14962 (PMC9828143; doi:10.1111/dme.14962)
Supplement: Supplementary file 1 — Figure S1 Figure S2 [file DME-39-0-s001.docx]

Supporting information:

***Supplementary figure 1:*** *Islet area was measured after haematoxylin and eosin staining of pancreatic sections. (A) Islet area in WT and KINGS male mice and (B) WT and KINGS female mice at 4, 10, and 20-weeks of age. Islets were imaged using a Zeiss Axiophot microscope at 10-20x magnification. Islets pooled from n=3, two-way ANOVA with Holm-Sidak post hoc, *p<0.05.*


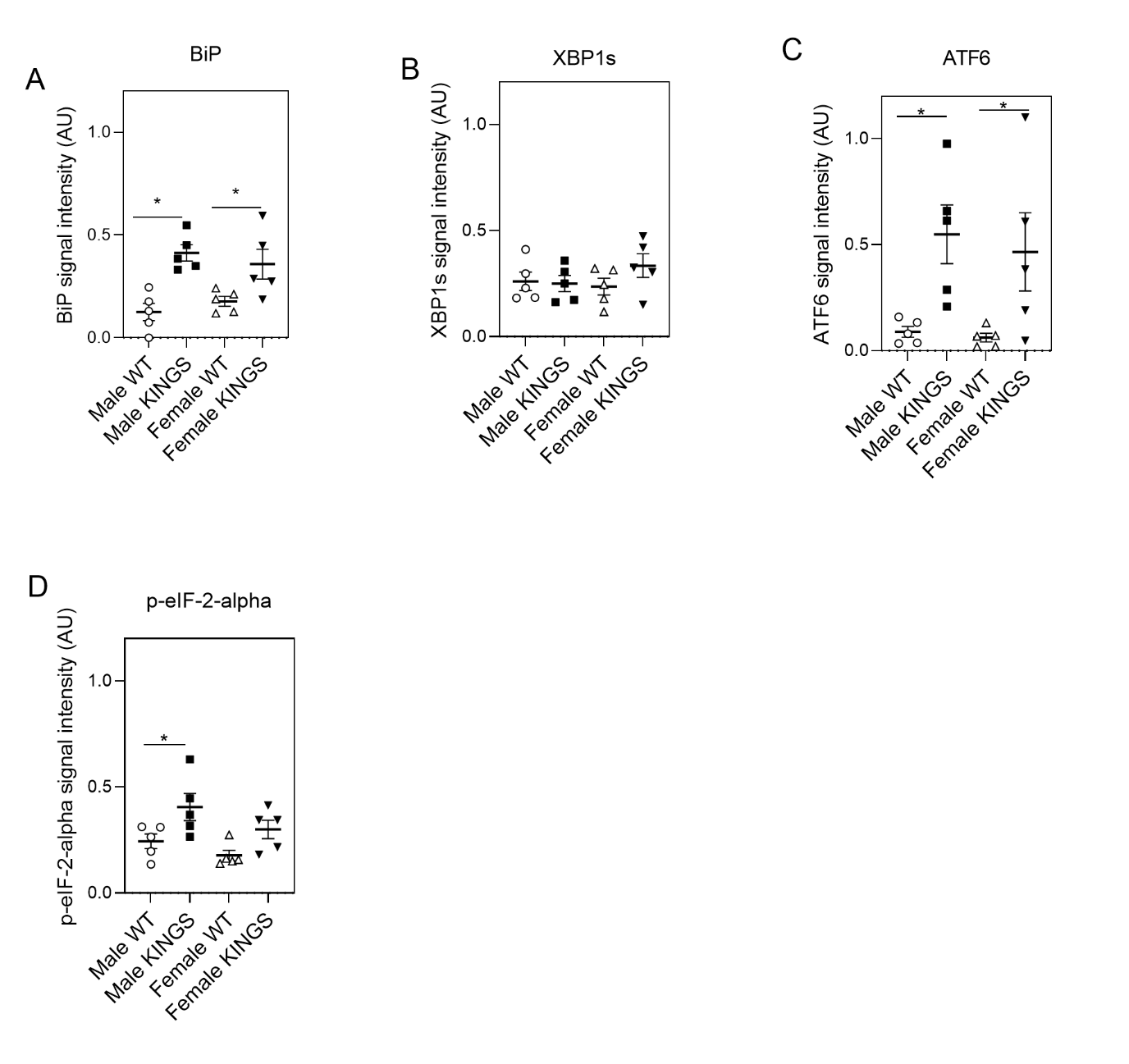


***Supplementary figure 2: Expression of UPR pathway markers in 4-week KINGS and wildtype mouse islets.*** *(A) BiP, (B) XBP1s, (C) ATF6, and (D) p-eIF-2-alpha was investigated in islets through western blotting. n=5, two-way ANOVA with Holm- Sidak’s post hoc, *P<0.05*
